# The role of mesenchymal stem cells derived exosomes as a novel nanobiotechnology target in the diagnosis and treatment of cancer

**DOI:** 10.3389/fbioe.2023.1214190

**Published:** 2023-08-17

**Authors:** You Zhou, Yuqing Dong, Aixue Zhang, Jibin Wu, Qiang Sun

**Affiliations:** ^1^ Department of Plastic Surgery, The First Hospital of China Medical University, Shenyang, China; ^2^ China Medical University and Department of Pathology, Shenyang, China

**Keywords:** mesenchymal stem cells, exosomes, biotechnology, cancer therapy, cancer diagnosis

## Abstract

Mesenchymal stem cells (MSCs), one of the most common types of stem cells, are involved in the modulation of the tumor microenvironment (TME). With the advancement of nanotechnology, exosomes, especially exosomes secreted by MSCs, have been found to play an important role in the initiation and development of tumors. In recent years, nanobiotechnology and bioengineering technology have been gradually developed to detect and identify exosomes for diagnosis and modify exosomes for tumor treatment. Several novel therapeutic strategies bioengineer exosomes to carry drugs, proteins, and RNAs, and further deliver their encapsulated cargoes to cancer cells through the properties of exosomes. The unique properties of exosomes in cancer treatment include targeting, low immunogenicity, flexibility in modification, and high biological barrier permeability. Nevertheless, the current comprehensive understanding of the roles of MSCs and their secreted exosomes in cancer development remain inadequate. It is necessary to better understand/update the mechanism of action of MSCs-secreted exosomes in cancer development, providing insights for better modification of exosomes through bioengineering technology and nanobiotechnology. Therefore, this review focuses on the role of MSCs-secreted exosomes and bioengineered exosomes in the development, progression, diagnosis, and treatment of cancer.

## 1 Introduction

Cancer is a life-threating disease and the leading cause of death in the world, with an estimated 19.3 million new diagnoses and 10 million deaths in 2020 (Sung et al., 2021). Benefiting from extensive research on cancer pathophysiology and molecular mechanisms, a variety of innovative approaches have been developed, such as targeted therapy, bacterial therapy, and immunotherapy (Waldman et al., 2020; Zhong L. et al., 2021; Gupta et al., 2021; Hou et al., 2022). Despite rapid advances in various approaches, most cancer remains incurable today. Many cancers develop drug resistance and continue to grow and metastasize, suggesting that effective control of tumor development and progression is still a long way off (Vasan et al., 2019; Saini and Twelves, 2021).

Current cancer diagnosis relies on symptoms, imaging, and biochemical indicators, all of which have various levels of sensitivity and specificity. Imaging examinations are usually not needed in individuals without symptoms of cancer, but the possibility of some cancers such as pancreatic cancer and colon cancer is easily overlooked because these cancers are often asymptomatic until they metastasize. Furthermore, benign or malignant nodules (e.g., thyroid nodules or lung nodules) are often inconclusive even with imaging examination and require further confirmation by invasive biopsy. Since delayed diagnosis leads to delayed treatments and a poorer prognosis (Aden et al., 2022; Barrios, 2022; Lone et al., 2022; Nicholson and Lyratzopoulos, 2023), detection of cancer-related biomarkers in a simple blood draw is the most important strategy for early detection and early treatment of cancer. Therefore, liquid biopsy is a recent trend in cancer screening and management (Handa et al., 2021; Lone et al., 2022) as it can quickly determine the levels of specific cancer-related biomarkers in body fluids, especially blood.

Cell-cell interactions in the tumor microenvironment (TME) play an important role in cancer initiation, progression, and metastasis. In addition to direct cell-cell interaction, these cancer cells, immune cells, and stromal cells present in the TME can also affect theire biological functions and phenotypes by secreting a variety of soluble factors through extracellular vesicles (EVs), such as exosomes and microvesicles (Walcher et al., 2020). EVs can carry macromolecules (e.g., DNA, proteins, RNAs, and lipids) to other cells in the TME, and can also reach distant sites to facilitate the formation of pre-metastatic niche. There is evidence that EVs in cancer cells contains distinct inclusions from normal cells, supporting the potential value of EVs as cancer biomarkers (van Niel et al., 2018; Shehzad et al., 2021). Of noted, engineered EVs can also be used as a delivery system to deliver specific substances to cancer cells (Elsharkasy et al., 2020).

Exosomes are a type of EVs with a bilayer membrane, which contain proteins, RNAs, lipids, metabolites, growth factors, cytokines, or other factors inside. Exosomes secreted by cells can deliver their cargo to other nearby cells or enter circulation to affect distant cells (Zhang Y. et al., 2020). Therefore, exosomes represent the current state of the cells that secrete them (Zhang Y. et al., 2020). Cancer cells are known to exhibit distinct transcriptomics and metabolomics from normal cells, and this difference can be reflected in the exosomes. Therefore, exosomes are attractive biomarker in liquid biopsy for early diagnosis of cancer and evaluation of treatment response (Handa et al., 2021; Lone et al., 2022). In addition to the potential use in cancer diagnosis, exosomes can be engineered to deliver payload consisting of microRNA (miRNA), small interfering RNA (siRNA), long non-coding RNA (lncRNA), proteins, and cytotoxic drugs to cancer cells (Walker et al., 2019; Dilsiz, 2020; Zhang et al., 2022).

Mesenchymal stem cells (MSCs), one of the main sources of stem cells in regenerative medicine, are often in the TME and play a critical promoting role in cancer development (Whiteside, 2018). MSCs are pluripotent cells with self-renewal capability and can also differentiate into osteoblasts, chondrocytes, and adipocytes. Given their potent immunomodulatory and immunosuppressive properties and tissue repair capabilities, MSCs have been considered as an attractive tool for the management of immune-related diseases such as inflammatory bowel disease and cancer (Kang et al., 2018). Inflammatory and immune responses in the TME are undoubtedly involved in cancer development (Greten and Grivennikov, 2019). Benefiting from immunosuppression and immune evasion in the TME, cancer cells continues to develop and progress under the surveillance of innate immunity (Kim and Cho, 2022). MSCs in the TME can not only produce extracellular matrix (ECM) components for cancer cells, but also secrete growth factors (Liang et al., 2021) to promote the polarization of M2 macrophages and expand myeloid-derived suppressor cells (MDSC), thereby facilitating immune evasion for cancer development (Ridge et al., 2017; Boutilier and Elsawa, 2021). The above regulation of cancer cells by MSCs is mainly achieved through paracrine activities, which is mediated by exosomes (Jafari et al., 2019).

Precision medicine provides breakthroughs in the detection of cancer-specific molecules and personalized precision therapy. Therefore, the purpose of this review is to comprehensively summarize the current application progress of MSCs-derived exosomes in cancer diagnosis and targeted therapy.

## 2 Structure and biogenesis of exosomes

EVs can be divided into three categories based on their size and biogenesis: 1) exosomes derived from exocytosis, with a size 30–200 nm; 2) microvesicles produced by budding and blebbing from the plasma membrane, with a size 100–1,000 nm, and; 3) apoptotic bodies released by apoptotic cells, with a size >1,000 nm (Colombo et al., 2014). Exosomes are membrane-bound extracellular vesicles that carry proteins, DNA, RNAs, and metabolites (Bobrie et al., 2011), and the internal content reflects the nature and the status of the cells that secrete them (Zamani et al., 2019). Under the electron microscopy, natural exosomes have a spheroid shape, while artificial or engineered exosomes exhibit a bi-concave or cup shape (Yellon and Davidson, 2014). The main biomarkers of exosomes include CD9, CD63, CD81, Alix, TSG101, integrins, heat shock proteins, actin, and flotillins (Zhang et al., 2019). The rigid bilayer membrane of the exosomes contains lipid components such as sphingomyelin, cholesterol, and ceramides, which are functionally involved in exosome secretion, structure, and signaling (Skotland et al., 2019). Various types of DNA and RNA are also commonly found in exosomes (Mashouri et al., 2019). Among them, miRNAs represent the most abundant RNA species in exosomes (Huang et al., 2013; Zhang et al., 2019) and are functionally involved in exosome-mediated cellular communication (Zhang et al., 2019).

**FIGURE 1:**
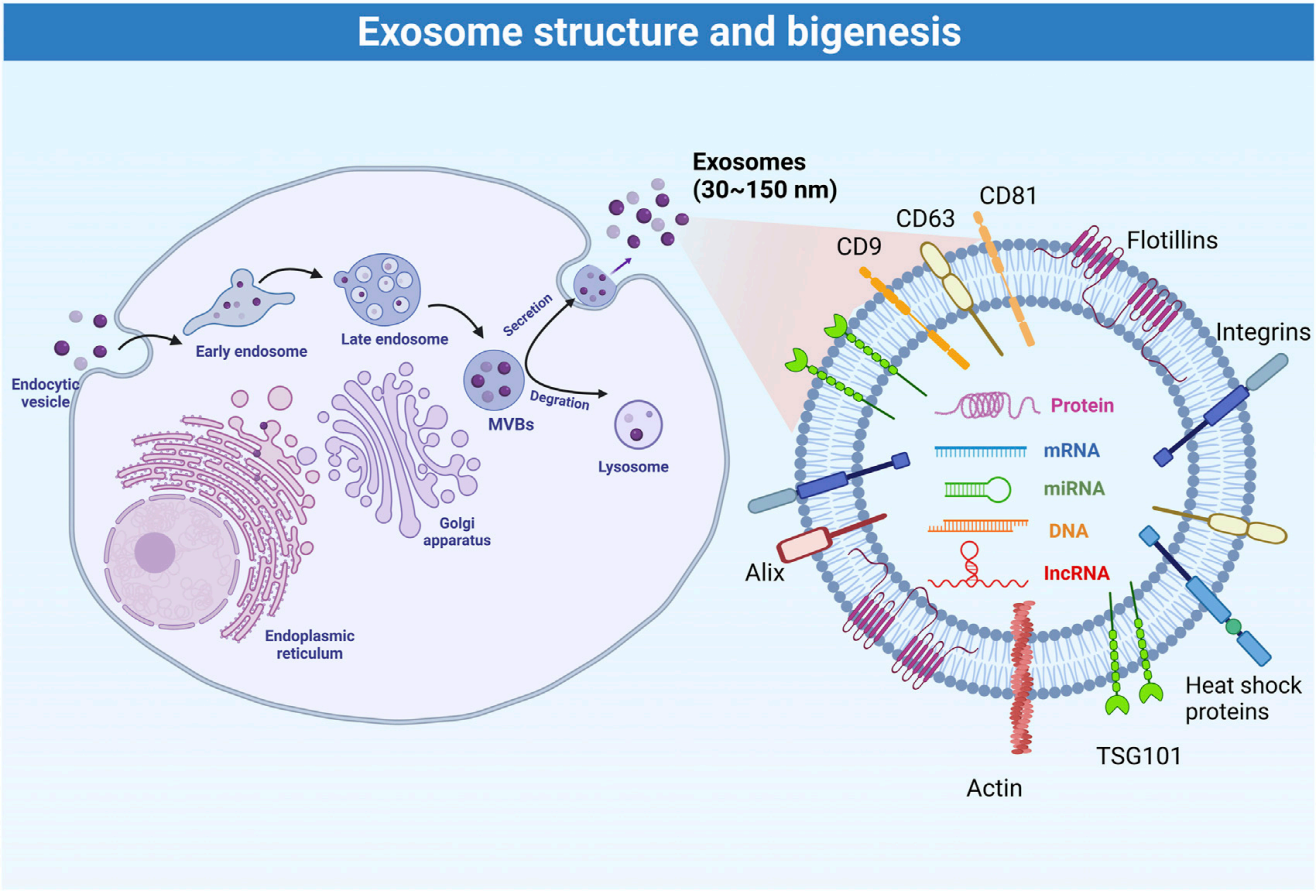
Schematic representation of the process of exosome biogenesis.

Figure 1 summarizes the process of exosome biogenesis. Multivesicular bodies and late endosomes are specialized endosomal compartments enriched in intraluminal vesicles that sequester specific proteins, lipids, and cytosolic components and are ultimately secreted as exosomes (Huang et al., 2013). Multivesicular bodies are transported by the cytoskeleton to the plasma membrane for exocytosis (Colombo et al., 2014), or to lysosomes or autophagosomes for degradation (Kalluri and LeBleu, 2020). Nevertheless, the molecular mechanisms regulating the secretion and/or degradation of multivesicular bodies remain poorly understood (White et al., 2006). It is also unclear whether specific transmembrane proteins or cargoes within the multivesicular bodies affect their secretion and degradation (Mashouri et al., 2019). It is now known that the biogenesis and secretion of intraluminal vesicles are driven by the endosomal sorting complex required for transport (ESCRT) machinery. ESCRT is a cytoplasmic multi-subunit system that is critical for membrane remodeling, multivesicular body sorting, and exosome secretion (Schmidt and Teis, 2012). Defects in any member of the ESCRT machinery may reduce exosome secretion (Colombo et al., 2013; Hoshino et al., 2013) or affect exosome composition (Baietti et al., 2012). Conversely, increasing the activity of ESCRT members such as the use of leptin could further increase exosome secretion (Giordano et al., 2019). Some viruses (e.g., hepatitis C virus) have also been found to affect the ESCRT machinery to promote the exosome-mediated transfer of viral RNAs (Dreux et al., 2012). Furthermore, ESCRT activity, exosome secretion, and exosome composition are also affected by ubiquitination (Putz et al., 2008), sphingolipid ceramide (Trajkovic et al., 2008), endosome-specific tetraspanins CD9, CD63 and CD81 (Perez-Hernandez et al., 2013), and Rab GTPases (Blanc and Vidal, 2018). Hence, the regulation of exosome secretion is a fine-tuned process that can respond to a variety of cellular and molecular factors. The board properties and characteristics of the exosome can be used to finely monitor physiological and pathological processes.

Exosomes are composed of various types of molecules, such as lipids on lipid bilayer (e.g., cholesterol, ceramides, sphingomyelin, phosphatidylinositol, phosphatidylserine, phosphatidylcholine, phosphatidylethanolamine, and gangliosides), glycoproteins (e.g., β-galactosidase, O-linked glycans, N-linked glycans), adhesion molecules (integrin-α, integrin-β, and P-selectin), tetraspanins (e.g., CD9, CD37, CD53, CD63, CD81, and CD82), antigen-presenting molecules (e.g., MHC Class I and MHC Class II), and other signaling receptors (e.g., FasL, TNF receptor, and TfR). These molecules are all involved in exosome biogenesis, cargo selection, secretion, release, targeting, and uptake. MHC molecules participate in the immune response through their antigen-presenting capabilities. Exosome components also include heat shock proteins (e.g., Hsp20, Hsp27, Hsp60, Hsp90, and Hsc70), cytoskeletal proteins (e.g., actin, cofilin, and tubulin), ESCRT machinery (e.g., Alix and TSG-101), and membrane transport and fusion proteins (e.g., GTPases, annexins, flotillin, Rabs, dynamin, and syntaxin), as well as a variety of growth factors, cytokines, and nucleic acids. Cargo within exosomes is thought to remain inert until delivered to the target cell, and then become active to regulate cellular metabolism (Gurung et al., 2021).

## 3 Mesenchymal stem cells (MSCs)

MSCs are adult stem cells with high differentiation potential and self-renewal capacity (Pittenger et al., 2019; Margiana et al., 2022). MSCs differentiation can be driven by a variety of factors, including cytokines, chemokines, extracellular vesicles, and inflammatory stimuli (Pittenger et al., 2019; Margiana et al., 2022). Benefiting from the characteristics of immune regulation and promoting cell survival, MSCs have been clinically used to treat various diseases such as metabolic abnormalities, inflammation, infection, immune disorders, and tissue injury damage (Yuan et al., 2021; Margiana et al., 2022). On the other hand, MSCs have dual characteristics of promoting and inhibiting tumorigenesis and progression. In generally, the innate immune system can recognize cancer cells and kill them. Therefore, a cancer cell must first escape the surveillance of the immune system before they grow into a mass of tumors (Prendergast, 2008), while the potent immunosuppressive function of MSCs helps cancer cells escape immune surveillance (Liang et al., 2021). In addition, MSCs are functionally involved in tumor angiogenesis, which is necessary to provide adequate nutrients to growing tumors (Li et al., 2016; Ghollasi et al., 2021). In aggressive tumors, cancer-associated fibroblasts (CAFs) can secrete a variety of pro-cancer cytokines and growth factors, which can further promote the differentiation of MSCs into CAFs (Kalluri, 2016). MSCs can also secrete growth factors to promote tumor development and invasion, which can serve as hallmarks of invasive cancer and metastatic spread (Wang and Zhou, 2013; Ribatti et al., 2020). Furthermore, MSCs can inhibit cancer stem cells (CSCs) apoptosis and promote their proliferation to further participate in cancer aggressiveness, drug resistance, metastasis, and recurrence (Walcher et al., 2020; Hayat et al., 2021).

In addition to promoting tumor growth, MSCs also have tumor suppressive properties (Ye et al., 2021). MSCs can suppress the proliferation of Kaposi’s sarcoma cells by inhibiting the activation of Akt signaling cascade (Khakoo et al., 2006), which is involved in the proliferation and survival of cancer cells (Madhunapantula et al., 2011). MSCs can also upregulate the expression of p21, a cell cycle inhibitor, in various cell lines including liver cancer, lymphoma, and insulinoma (Lu et al., 2008). Besides, MSCs can increase the infiltration of inflammatory cells, regulate cancer cell cycle, and inhibit angiogenesis (Chen X. et al., 2021). Nevertheless, the balance of the dual roles of MSCs on cancer cells and their functional switch remains to be elucidated.

## 4 Regulatory mechanisms of MSCs-derived exosome in cancer

Regarding the role of EVs in cancer, Zhu et al. (Zhu et al., 2012) showed that exosomes secreted by MSCs had the same angiogenesis-promoting effects as MSCs themselves in a xenograft mouse model of gastrointestinal cancer. A study by Lee et al. (Lee et al., 2013) reported an opposite role of MSCs in breast cancer cells and found that this difference may be attributed to the amount and types of miRNAs in exosomes, which was further supported by Pakravan et al. (Pakravan et al., 2017) and other studies (Alcayaga-Miranda et al., 2016; Rosenberger et al., 2019). In other words, MSCs exert their dual functions to enhance or inhibit tumor development in a paracrine manner (Ridge et al., 2017; Kang et al., 2018), which is closely related to tumor-associated miRNAs contained in MSC-derived exosomes. Several studies have investigated the effects of specific miRNAs in exosomes on cancer cells (Dilsiz, 2020; Li B. et al., 2021; Li et al., 2022). MSC-derived exosomes can promote tumor development in a variety of solid tumors through their internal miRNAs (Du et al., 2014; Zhao et al., 2019; Zhou et al., 2019), which appears to be associated with the activation of the extracellular signal-regulated kinase 1/2 (ERK1/2) pathway (Zhu et al., 2012). Furthermore, several miRNAs, proteins, and long non-coding RNAs (lncRNAs) in MSC-derived exosomes were found to suppress the cancer cell proliferation and promote cancer cell apoptosis (Bruno et al., 2013; Wu et al., 2013; Furuta et al., 2016; Reza et al., 2016; Takahara et al., 2016; Maffey et al., 2017; Zhang F. et al., 2020).

Cancer cells can invade surrounding tissues through three main patterns: amoeboid cell migration, mesenchymal cell migration, and collective cell migration. Moreover, TME and anticancer drugs can switch the invasive pattern of cancer cells, resulting in limited efficacy of anticancer drugs (Wu et al., 2021). From a cellular perspective, epithelial-to-mesenchymal transition (EMT) is a critical step in the invasion and metastatic spread of solid tumors. After EMT, cancer cells acquire migration and invasion capacities and reduce their adhesion to surrounding cells (Wang and Zhou, 2013; Ribatti et al., 2020). Mesenchymal cells are elongated cells that can move forward using cytoskeletal contractility (Wu et al., 2021). Studies have shown that MSC-derived exosomes can regulate EMT through the ERK pathway (Du et al., 2016; Maffey et al., 2017). In addition, exosomes secreted by MSCs can promote the transformation of macrophages into cancer-associated macrophages, thereby promoting EMT, cancer cell proliferation, migration and invasion, and distant tumorigenesis (Paskeh et al., 2022; Du et al., 2014; Shi et al., 2016; Dong et al., 2018; Zhao et al., 2019; Zhou et al., 2019). MSC-derived exosomes have also been reported to be involved in tumor dormancy (Lugano et al., 2020; Zheng X. et al., 2021; Zhong Y. et al., 2021), which is the ability of tumors to remain in a small number of undetectable tumor cells after primary tumor resection. Notably, tumor dormancy is associated with anticancer drug resistance, prolonged asymptomatic residual disease, and cancer recurrence (Gomis and Gawrzak, 2017). Breast cancer cells that migrated to the bone marrow were found to hide within MSCs populations and become dormant and chemoresistant until reactivated (Fornetti et al., 2018).

Resistance of cancer cells to anticancer drugs is a great challenge in the clinical treatment of cancer. Tumor cells can acquire resistance to specific drugs through mutations, polymorphisms, and splicing variations in the various genes during evolution process in response to drug toxicity or targeted metabolism (Lin et al., 2020). One of the most common resistance mechanisms is the overexpression of membrane transporters that actively pump these absorbed anticancer drugs out of cancer cell. Another mechanism is that the targets of anticancer drugs are mutated to reduce drug efficacy and toxicity. Anticancer drug resistance also includes the activation of mechanisms that favor cancer cell survival and decrease apoptosis (He et al., 2020). Overactivation of DNA repair mechanisms can also reduce the effectiveness of anticancer drugs that cause DNA damage (Zheng X. et al., 2021). Importantly, exosomes are involved in all of these mechanisms of anticancer drug resistance (Zheng X. et al., 2021; Zhong Y. et al., 2021). Another feature of tumor development is angiogenesis, which is a necessary process to ensure adequate nutrient and oxygen supply during cancer cell growth (Lugano et al., 2020). Nonetheless, the role of MSC-derived exosomes in angiogenesis remains controversial. Some studies reported that MSC-derived exosomes can induce angiogenesis through the Wnt pathway (Salomon et al., 2013; Lopatina et al., 2014; Gong et al., 2017; McBride et al., 2017; Olsen et al., 2017). However, some studies reported different findings that MSC-derived exosomes can reduce angiogenesis by downregulating VEGF and CD31 (Lee et al., 2013; Pakravan et al., 2017).

In addition to functioning as carriers of proteins, RNAs, and cytokines, exosomes can also act as antigen carriers to stimulate innate and adaptive immune responses (Thery et al., 2009). IDO-1-containing exosomes secreted by MSCs can reduce IFN-γ expression in dendritic cells and NK cells (He et al., 2018). Furthermore, MSC-derived exosomes can also induce IL-10 secretion, increase the numbers of regulatory T (Treg) cells, and suppress Th17 activation, thereby promoting immune escape (Favaro et al., 2016).

## 5 The role of mesenchymal stem cell derived exosomes RNA in cancer diagnosis and prognosis

Needle biopsies and surgical excision biopsies are most common methods to obtain cancer biospecimens. A needle biopsy is a procedure to obtain a small portion of cells, which may miss aggressive or biologically different lesions in case of tumor heterogeneity. Although surgical excision biopsy can examine the entire tumor, it cannot provide detailed information on disseminated tumor cells and metastases. Another concern is that the biopsy procedure is invasive, with potential risks of complications such as discomfort, hemorrhage, and infection. In contrast, liquid biopsies have received increasing attention in cancer diagnosis and monitoring due to the advantage of requiring only a simple blood draw (Handa et al., 2021; Lone et al., 2022). Liquid biopsies based on the detection of circulating cancer cells, miRNAs, EVs, cell-free DNA, and proteins. The advantages of liquid biopsy are that biomaterials can be obtained rapidly and easily, with minimal pain and risk for patients, and it also allows a comprehensive assessment of tumor burden. Moreover, liquid biopsy has several limitations, including challenging in the isolation of biomaterials, short half-life of the biomarkers in the biomaterials, and possible contamination by normal cells (Handa et al., 2021; Lone et al., 2022). Compared with other materials obtained by liquid biopsy, exosomes are of interest because they contain miRNAs derived from cancer cells and MSCs. Since specific miRNAs may be upregulated in cancer cells, monitoring specific miRNAs is an effective strategy for cancer screening, diagnosis, and monitoring (Handa et al., 2021; Lone et al., 2022). In addition, exosomal miRNAs are present in all human physiological fluids, such as plasma, serum, urine, saliva, bile, breast milk, and cerebrospinal fluid. Given the prevalence of exosomes in physiological fluids and the stability of miRNAs in exosome, it is feasible and practicable to use exosomal miRNAs as unique biomarkers for early cancer diagnosis.

The number of detectable exosomes is increased in cancer patients compared with healthy controls (Suchorska and Lach, 2016). Therefore, exosomal RNA has been used for the diagnosis and prognosis of various cancers (Zhu et al., 2019). For example, the levels of miR-221 in peripheral blood has been suggested as a diagnostic marker for gastric cancer (Huang et al., 2018). Increased levels of exosomal miR-214, miR-221, and miR-222 are associated with the development of gastric cancer (Wang et al., 2014), and upregulation of miR-214 is associated with venous invasion and poor prognosis of gastric cancer (Ueda et al., 2010). In addition, miR-122 is associated with the diagnosis and prognosis of liver cancer (Lu et al., 2008; Coulouarn et al., 2009), while miR-1231 has diagnostic value for pancreatic cancer (Shang et al., 2019). Table 1 lists several dysregulated miRNAs in cancer cells, and Table 2 lists several miRNAs that have been confirmed to have accurate cancer diagnostic performance.

**TABLE 1:** miRNAs associated with cancer.

Cancer type	miRNA
Brain	miR-7
	miR-101
	miR-29a/b/c
	miR-146b-5p
	miR-181c
	miR-320a
	miR-21
	miR-221, miR-222
	miR-10b
	miR-181b
	miR-141
Head & neck	Let-7c
	miR-101
	miR-124
	miR-let-7e
	miR-206
	miR-30a, miR-379
	miR-125a
	miR-134
	miR-196b
	miR-144
Breast	miR-126
	miR-204
	miR-720
	miR-205
	miR-200
	miR-203a-3p
	miR-1-3p
	miR-210
	miR-182
	miR-155
	miR-526b, miR-655
	miR-20b
	miR-155, miR-203, miR-125a
Gastrointestinal	miR-28-5p
	miR-7
	miR-1299
	miR-223-3p
	miR-339-5p
	miR-148a-3p, miR-181a-5p
	miR-497
	miR-100
	miR-181a
	miR-653-5p
	miR-1301-3p
	miR-106a, miR-18a, miR-20b, miR-486-5p, miR-584
	miR-34a-5p
	miR-199a-3p
	miR-103, miR-720
	miR-19a-3p, miR-19b-3p, miR-25-3p, miR-195-5p, miR-223-3p
Genitourinary	miR-199a-3p
	miR-203
	miR-218
	miR-1
	miR-31-5p
	miR-381
	miR-125b
	miR486-5p
	miR-4534
Gynecologic	Led-7d-5p
	miR-101-5p
	miR-132
	miR-138-5p
	miR-148b
	miR-508, miR-509-2, miR-526b
	miR-16-1
	miR-20a
	miR-20b
	miR-27b
	miR-106b-5p

**TABLE 2:** miRNAs studied for cancer diagnostic accuracy.

Cancer type	miRNA
Colorectal	miR-223, miR-92a
	miR92a, miR-144
	miR-24, miR-320a, miR-423-5p
	miR-1246, miR-202-3p, miR-21-3p, miR-1229-3p, miR-532-3p
	miR-15b, miR-21, miR-31
	miR19a, miR19b, miR15b, miR29a, miR335, miR18a
	miR-29a, miR92a
	miR-21-5p, miR-1246, miR-1229-5p, miR-96-5p
	miR-431, miR-139-3p
	miR-506, miR-4316
	miR-27a, miR-130a
	miR-30e-3p, miR-146a-5p, miR-148a-3p
	miR-186-5p, miR-29a-3p, miR-29c-3p, miR-766-3p, miR-491-5p
	miR-144-3p, miR-425-5p, miR-1260b
	miR-23a-3p, miR-27a-3p, miR-142-5p, miR-376c-3p
	miR-601, miR-760
	miR-7, miR-93, miR-409-3p
	miR-18a, miR-21, miR-22, miR-25
	hsa-miR-451a, hsa-miR-144-5p, hsa-miR-200b-3p
	miR-103a-3p, miR-127-3p
	miR-151a-5p, miR-17-5p, miR-181a-5p, miR-18a-5p, miR-18b-5p
	Panel of 19 miRNAs
	miR-139-3p
	miR-126, miR-1290, miR-23a, miR-940
	miR-1246, miR-1290, miR-4323, miR-4284
Lung	miR-1268b, miR-6075
	miR-21-5p, miR-141-3p, miR-126-3p, miR-146a-5p, miR-222-3p, miR-223-3p, miR-155-5p, and miR-486-5p
	miR-125b-5p, miR-5684
	miR-620
Liver	miR-211
	miR-16
	miR-19a, miR-296, miR-195, miR-192, miR-34a
	miR-122, miR-244
	miR-101-1, miR-221
	miR-214-5p, miR-494, miR-138b, miR-125b, miR-1269, miR-145, miR-375
	miR-182, miR-150
	miR-27a, miR-18b, miR-301
	miR-215
	miR-23b-3p, miR-331-3p
	miR-125a-5p
	miR0let-7a-1
Prostate	miR-141
	miR-141-3p, miR-125a-5p
	miR-486-5p, miR-451a, miR-486-3p, miR-375
Ovarian	miR-200a/b/c
	miR-200c, miR-141
Endometrial Breast	miR-200-a, miR-141
	miR-200a/b/c, miR-141
	miR-429
	miR-21-5p
	miR-1910-3p
	miR-17-5p
	miR-423, miR-424, let7-I, miR-660
	miR-3662, miR-146a, miR-1290
Stomach Oral	miR-200c
	miR-24a-3p
	miR-130a
	miR-155, miR-21

Lung cancer, one of the most common and deadly forms of cancers in the world, is often diagnosed at advanced stages (Sung et al., 2021). Wu et al. (Wu et al., 2020) identified a panel of eight exosomal miRNAs that can effectively detect stage I or II lung cancer with high accuracy. Li et al. (Li X. et al., 2021) found that miR3913-5p levels were associated with increased treatment resistance. The levels of miR-125b-5p and miR-5684 have diagnostic and prognostic values in lung cancer (Zhang Z. et al., 2020), while the level of miR-620 levels is significantly lower in patients with lung cancer (Tang et al., 2020). Furthermore exosomal miR-1246 was reported to be significantly associated with TNM stage (Huang and Qu, 2020), while a panel of six miRNAs was found to be associated with radioresistance (Zheng Q. et al., 2021).

Breast cancer is the most common cancer in women in the world and (Sung et al., 2021). Several miRNAs were identified as potential biomarkers for the diagnosis of breast cancer, such as miR-1910-3p (Wang et al., 2020), miR-17-5p (Lv et al., 2020), and a panel of four urinary exosomal miRNAs (Hirschfeld et al., 2020). Studies have shown that the sensitivity and specificity of exosomes in the diagnosis of breast cancer are 93% and 87%, respectively (Liu M. et al., 2021). In addition, some miRNAs have also identified to have prognostic value in breast cancer (Li D. et al., 2020; Wang X. et al., 2021; Xun et al., 2021).

With regard to prostate cancer, miRNAs in serum and urinary exosomes have also been identified as having diagnostic (Li W. et al., 2020; Li Z. et al., 2021) or prognostic (Guo et al., 2020; Kim et al., 2021; Rode et al., 2021) values. In addition, several miRNAs have been identified for use in the diagnosis and prognosis of various types of cancer (e.g., oral squamous cell carcinoma and colorectal cancer) (Preethi et al., 2022). Since serum contains a variety of exosomes in circulation, only one blood draw can simultaneously detect the profile of multiple miRNAs, which represent the characteristics of different tumors. In a clinical sense, detection of miRNAs from circulating exosomes could serve as a useful screening tool for cancer diagnosis to start early treatment strategies.

EVs can also be used to deliver short peptides to targeted cancer cells for cancer therapy. For example, delivery of GSK-J1 has good effects on carboplatin-resistant ovarian cancer showed promising therapeutic efficacy, including induction of cancer cell apoptosis, reduction of cell motility, and prevention of cell spheroids (Yang et al., 2022). Even for difficult-to-treat glioblastoma with poor prognosis, several exosome-based platforms have been established to overcome the blood–brain barrier (BBB) and showed promising results, such as zinc sulfide-based hybrid exosome-coated nanoplatform and HDX@YSN @ CCM@cRGD delivery system (Mo et al., 2022; Liu et al., 2023).

## 6 Therapeutic applications of MSC-derived exosomes

Since exosomes are involved in the paracrine signaling, MSC-secreted exosomes secreted by MSCs will be a good tool for cancer treatment. In fact, some studies have reported the promising results of exosomes in cancer therapy (Staufer et al., 2021; Ferreira et al., 2022). It still need to pay attention that MSCs themselves and the exosomes they secrete have dual characteristics that promote and inhibit cancer development (Weng et al., 2021). Nevertheless, exosomes have interesting characteristics for cancer therapy, such as targeting, low immunogenicity, modification flexibility, and high BBB permeability (Dalmizrak and Dalmizrak, 2022).

Various methods are currently available for purifying exosomes (Thery et al., 2018; Wang J. et al., 2021), but none guarantee the isolation of pure exosomes, and often require further identification and purification steps (Thery et al., 2018). Despite these effective methods, expensive equipment and large samples are necessary. In addition, and each step has a risk of contamination, resulting in low efficiency, high sample loss, and low exosome recovery rate, and low purity. The common methods used for exosome purification include gold standard ultracentrifugation, density gradient centrifugation, size exclusion chromatography, immunoaffinity, and polymer precipitation. Some novel techniques that only require smaller samples, shorter purification time and higher recovery efficiency have been developed, including TiO2-based exosome isolation, Fe3O4@TiO2-CD63 aptamer, ExoCAS-2, microvortex chips, acoustofluidic platform, acoustofluidic centrifugation, paper-based anionic isotachophoresis, microfluidic nanowire array, ExoDFF, raman assay chip, and lipid microarray (Chen J. et al., 2021). Although these new methods require specific advanced equipment, they are promising approaches to obtain high-purity exosomes.

Several studies investigated the effects of exosomes secreted by MSCs on different types of cancer cells *in vitro* and *in vivo*. In breast cancer, MSC-secreted exosomes carrying miR-16 can inhibit angiogenesis and tumor progression (Lee et al., 2013), and exosomes carrying miR-100 can inhibit angiogenesis as well as tumor proliferation and migration (Pakravan et al., 2017). In breast cancer metastatic to the bones, MSC-derived exosomes carrying miR-23b not only reduced tumor proliferation and invasion, but also increased dormancy of metastatic cancer cells and decreased sensitivity to docetaxel (Ono et al., 2014). In prostate cancer, MSC-derived exosomes carrying miR-145 reduced proliferation and promote apoptosis of cancer cells (Takahara et al., 2016). In lung cancer, let-7i-loaded exosomes have been demonstrated to inhibit proliferation and metastasis of lung cancer cells (Liu J. et al., 2021). In a phase I clinical trial, autologous ascites-derived exosomes combined with GM-CSF can induce immune response against colorectal cancer (Dai et al., 2008).

Exosomes can be isolated and purified from a variety of cells, and additional engineering of these exosomes further increases the therapeutic potential. Exosome engineering includes cargo/payload and surface engineering. Cargo/payload engineering allows the encapsulation of specific molecules (e.g., proteins, miRNA, lncRNA, etc.) within exosome. Furthermore, therapeutic drugs can also be loaded in the hydrophilic core or the lipophilic membrane of the exosomes (Haney et al., 2019; Walker et al., 2019). Surface modification engineering of exosomes can render them more targeted toward specific cells, especially different cancer cells (Liang et al., 2021). In other words, these exosomes loaded with RNAs, proteins, drugs, and other chemicals can target specific cells or cancer cells to exert anticancer effects. The engineering of exosomes mainly includes two strategies: engineering before exosome isolation and engineering after exosome isolation (Weng et al., 2021).

In the “engineering before exosome isolation” strategy, the exosome-providing cells are first modified to package interest therapeutic cargo in exosomes. A common strategy is to overexpress therapeutic RNAs and/or proteins, resulting in these overexpressed RNAs/proteins being encapsulated in exosomes (Herrmann et al., 2021). Another strategy is to incubate these exosome-providing cells with drugs to generate drug-loaded exosomes. Therefore, engineering before exosome isolation strategy usually preserves the native membrane of exosomes (Weng et al., 2021). An ongoing early phase I trial is investigating the clinical application value of personalized vaccines made from exosomes derived from patient-isolated tumor cells, dendritic cells, and macrophages in patients with recurrent/metastatic bladder cancer (ClinicalTrials.gov identifier: NCT05559177).

In the “engineering after exosome isolation” strategy, the isolated exosomes are passively or actively loaded with payloads. Lipophilic drugs can be passively absorbed by exosomes in a concentration gradient manner, while hydrophilic drugs can be loaded to exosomes by electroporation, sonication, freeze/thaw cycles, extrusion, and chemicals (Weng et al., 2021). Nonetheless, caution must be taken to avoid exosome aggregation, membrane damage, or loss of immunogenicity. Several new technologies are also being developed, such as the EXPLORs strategy to encapsulate anti-inflammatory peptides in exosomes (Yim et al., 2016), and protein-based sorting of miRNAs into exosomes (Villarroya-Beltri et al., 2013; Shurtleff et al., 2016). Surface molecules on the exosomal membrane can affect the selectivity of exosomes for specific target cells. Hence, modifying the surface molecules of exosomes can alter the biodistribution and tropism of the exosomes. In fact, the main goal of surface engineering is to increase the specificity of exosomes to specific targets, most of which target cancer cells to protect normal cells and reduce systemic treatment toxicity. Surface engineering is usually achieved through genetic engineering, chemical modification, and hybrid membrane engineering (Weng et al., 2021). Genetic engineering involves transfecting cells with plasmids to overexpress RNAs or proteins of interest, indirectly promoting exosome loading. For example, increasing the N-terminal portion of Lamp2b protein on the surface of exosomes can increase the binding affinity and selectivity for ligands (Alvarez-Erviti et al., 2011; Weng et al., 2021). Nevertheless, genetic engineering of exosome modification still has some questions to be solved. The main questions include the correct expression of the fusion protein, the accuracy of target recognition, the possibility of loss of function, and the loss of immunogenicity. The strategy of chemical modification involves the covalent bonding of molecules to the surface of exosomes to target specific cells.

The ultimate goal of exosomes in cancer therapy is to precisely deliver cargo or payload to cancer cells, thereby reducing cancer cell proliferation and invasiveness, promoting cancer cell death, and/or increasing sensitivity to other therapeutic drugs. Regarding miRNAs as cargo for exosomes, encapsulation of miRNAs in exosomes is an suitable delivery strategy because free miRNAs are easily degraded in circulation (Zhang et al., 2022). There are two miRNA-based strategies for cancer therapy: miRNA suppression and miRNA replacement. The miRNA suppression strategy is used when the target miRNA suppresses the oncogene, while the miRNA replacement strategy can be used when the miRNA to be replaced is downregulated in the cancer cells and cannot inhibit the oncogene (Dalmizrak and Dalmizrak, 2022; Zhang and Farwell, 2008). Given the complexity of cancer mechanisms, simultaneous targeting of multiple genes is permissible (Baumann and Winkler, 2014). Furthermore, the advantage of using miRNAs as therapeutic tools is that most miRNAs can target and regulate multiple genes simultaneously (De Veirman et al., 2016; Li and Li, 2018). For example, exosomes carrying miR-122 can reduce the proliferation and increase the sensitivity of liver cancer cells to 5-fluorouracil (5-FU) and doxorubicin (Fornari et al., 2009; Zhang et al., 2013; Li X. et al., 2020). Exosomes loaded with miR199a-3p can downregulate the expression of YAP1, CD151, and mTOR and increase chemosensitivity (Fornari et al., 2010; Kim et al., 2016; Ren et al., 2016). Exosomes loaded with miR-379 suppress breast cancer growth by regulating COX-2 (O'Brien et al., 2018). In glioma, exosomes carrying miR-146b, miR-124a, or miR-34a have been shown to decrease tumor proliferation by inhibiting EGFR, NF-κB, FOXA2, and MYCN (Katakowski et al., 2013; Lang et al., 2018; Wang B. et al., 2019). Besides, many studies have also revealed the effects of miRNAs in exosomes in various cancers, such as breast cancer (O'Brien et al., 2018; Yuan et al., 2019), glioma (Katakowski et al., 2013; Lang et al., 2018), glioblastoma (Wang B. et al., 2019), colorectal cancer (Xu et al., 2019), prostate cancer (Jiang et al., 2019), endometrial cancer (Li et al., 2019), pancreatic cancer (Wu et al., 2019), cervical cancer (Zhang H. et al., 2020), and ovarian cancer (Meng et al., 2021). Table 3 lists several miRNAs with cancer therapeutic value. There is no doubt that all current studies support the effectiveness of exosomes in delivering miRNAs to fight cancer. However, there is a potential safety concern regarding exosome-based miRNAs delivery before clinical application.

**TABLE 3:** miRNAs studied for cancer treatment.

Cancer type	miRNA
Glioma	miR584
	miR133b
	miR34a
	miR-199a
Glioblastoma	miR-124
	miR-4731
	miR-512-5p
	miR30c
Neuroblastoma	miR-124
Oral cancer	miR-101-3p
Thyroid cancer	miR-30c-5p
Breast cancer	LNA-antimiR-142-3p
	miR-148b-3p
	miR-145
	miR-3182
	miR-381
Esophageal cancer	miR-375
Gastric cancer	miR-6785-5p
Pancreatic cancer	miR-145-5p
Liver cancer	miR-122
	miR-199a
Prostate cancer	miR-205
Bladder cancer	miR-139-5p
Endometrial cancer	miR-302a
Cervical cancer	miR-144-3p
Ovarian cancer	miR-424
Bone cancer	miR-143
	miR-9-5p
Lung cancer	miR-328-3p
	miR-320a

In addition to miRNAs being attractive payloads for cancer therapy, other payloads also have potential. Zhou et al. (Zhou W. et al., 2021) proposed the use of MSC-derived exosomes to deliver oxaliplatin and siRNAs for the treatment of pancreatic cancer. Paclitaxel is a highly hydrophobic compound, and its traditional formulation relies on solvents and excipients known to cause toxicity (Fu et al., 2018; Oun et al., 2018). In addition, exosome-encapsulated paclitaxel could also decrease its treatment-related cytotoxicity (Wang P. et al., 2019). The effectiveness of paclitaxel-loaded exosomes has been confirmed in various cancers, such as pancreatic, breast, lung, and ovarian cancers using is also possible (Pascucci et al., 2014; Melzer et al., 2019). In particular, exosomes loaded with doxorubicin can cross the BBB, which is the main reason why brain metastases are difficult to treat (Yang et al., 2015). Besides, exosomes can effectively deliver other anticancer drugs, such as porphyrin, tirapazamine, docetaxel, and cisplatin (Zhang Y. et al., 2020). An attractive feature of exosome-based drug delivery system is that it is not limited to the intravenous route of administration, as subcutaneous, intraperitoneal, intratumoral, intranasal, and oral routes are also potential routes (Zhang Y. et al., 2020). An ongoing phase I clinical trial was conducted to investigate the effect of plant (grape)-derived exosomes loaded with curcumin for the treatment of colon cancer (ClinicalTrials.gov NCT01294072), but the step of engineered MSC-derived exosomes is still in progress.

## 7 Clinical applications of MSC-derived exosomes

**FIGURE 2:**
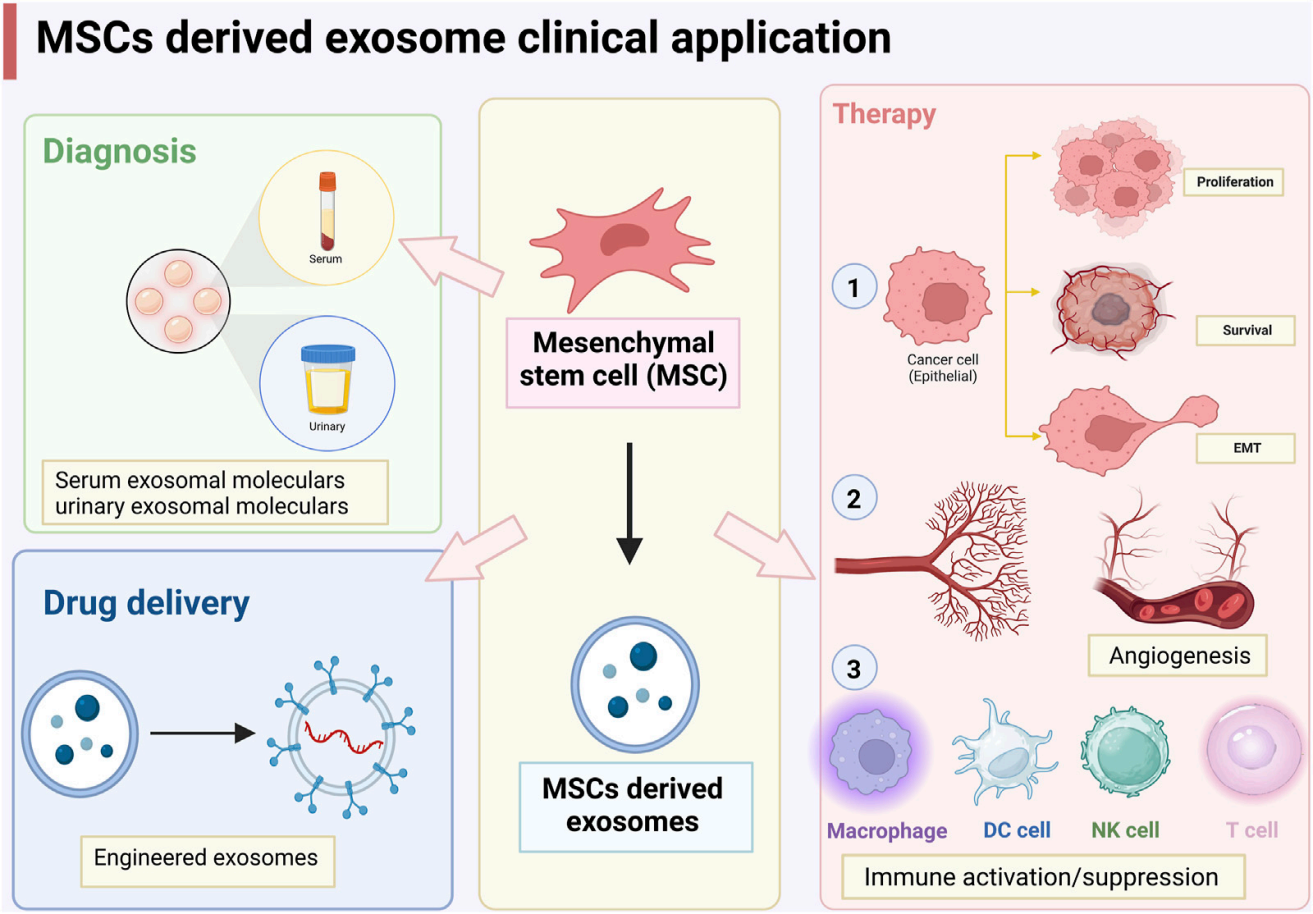
Clinical applications of mesenchymal stem cells (MSCs)-derived exosomes.

As mentioned above, MSC-derived exosomes can promote and/or inhibit cancer cell proliferation and apoptosis, EMT, angiogenesis, and immune activation (Figure 2). The dual role of MSCs in cancer development are mainly attributed to different proteins, miRNAs, lncRNAs, and cytokines encapsulated in exosomes. Challenges in using MSC-derived exosomes for clinical diagnosis and therapy include efficient and rapid exosome isolation techniques, long-term preservation of exosomes, and rapid quantification and identification of exosomes. In addition, the use of exosomes for therapeutic purposes also involves the large-scale production of engineered exosomes, the high specificity in targeting cancer cells, long-term safety, and the property of avoiding macrophage phagocytosis and destruction (Weng et al., 2021). Although all cells can secrete exosomes, MSCs are the most prolific exosome producers commercially and have been approved by the FDA for therapeutic purposes (Zhou T. et al., 2021). In addition to the advantage of modification and storage, MSC-derived exosomes are natural carriers, which are more biocompatible and less immunogenic than other nanocarriers such as liposomes (Weng et al., 2021). Therefore, MSC-derived exosomes have great potential in cancer therapy.

Current technologies for producing exosomes are insufficient to meet clinical needs and further improvement are needed, including reliable large-scale production, qualified and consistent exosome content, and avoidance of contamination. First of all, increasing the yield of MSCs from cell culture is the most important part. Currently, MSCs are usually cultured in two-dimensional plastic tissue Petri dish and flasks, which greatly limit the area for their growth. After all, the two-dimensional growth environment is not comparable to the three-dimensional environment in the body. Therefore, some synthetic biomaterial scaffolds have been designed to mimic the structure and function of the ECM. Some studies have shown that Avitene Ultrafoam collagen hemostat doubles the yield of exosomes released by BM-MSCs compared with those cultured in plastic tissue Petri dish (Tao et al., 2017). Second, the production of exosomes can be improved by regulating the lysosome pathway to affect the production, secretion and degradation process of exosomes. For example, activation of P2X7 receptors on the membrane can enhance exosome production by triggering membrane blistering, sorting of endosomal contents, and fusion with polyvesicles to accelerate exosome release (Qu and Dubyak, 2009).

In recent years, some studies have focused on methods for efficient and large-scale production of exosomes. Yang Zhaogang et al. developed a method of cell nanoporation that can generate large numbers of functional mRNA-encapsulating exosomes. Compared with other strategies for producing EVs, batch electroporation produced higher yields. Cellular nanoporation produced up to 50-fold increase in exosomes and more than 1000-fold increase in exosomal mRNA transcripts. Similar excellent results were also observed in cells with low basal levels of exosome secretion (Yang et al., 2020).

## 8 Application of exosome mimics as substitutes for MSCs-derived exosomes in cancer therapy

Despites the promise of exosomes as a drug delivery method in cancer therapy, the large-scale production of exosomes remains a challenging issue. In addition, the drug delivery efficiency of exosomes is also an unresolved problem. Once in the body, exosomes are easily cleared, and the therapeutic effect will be greatly reduced, especially for exosomes with low drug loading (Chen C. et al., 2021). Therefore, exosome mimetics (EMs), as a substitute for natural exosomes, are gradually becoming a more promising drug delivery platform due to their higher yields and the same biological functions as exosomes. EMs have many advantages as carriers for cancer treatment. First, the lipid bilayer of EMs can also fuse with the cell membrane, facilitating the internalization of packaged drugs. Second, the size of the EMs can be adjusted, which facilitates their infiltration into tumor blood vessels and spread into tumor tissue (Guo et al., 2021; García-Fernández and Fuente Freire, 2023).

Structurally, EMs have the same lipid bilayer structure as exosomes. In addition, EMs can utilize protein functionalized vesicle surfaces to regulate target cells to increase their circulation in the blood either by direct contact or by connecting hydrophilic molecules on the vesicle surface. EMs are mainly divided into liposomes, exosome-liposome nanoparticle hybrid system, exosome-inorganic/organic nanoparticle hybrid system, etc.

Liposomes, one of the most widely studied artificial EMs, are characterized by high permeability and retention. Therefore, liposomes can accumulate in tumors through vascular system. In addition, PEGylation based liposomes can reduce their interactions with blood proteins and immune cells, preventing them from being coated with blood proteins, recognized as foreign particles, and then cleared by macrophages (Mohamed et al., 2019). Currently, there are some marketed liposomal drug products as delivery systems, such as Inflexil ^®^ V. Doxil ^®^, Lipusu ^®^, and DaunoXOme ^®^. In addition, some are already in clinical trials, such as Amikacin liposomes (Beltrán-Gracia et al., 2019; Boutilier and Elsawa, 2021).

Numerous studies have been conducted on the design of exosome-liposome hybrid structures to improve drug delivery systems. The extrusion method is the simplest and most effective method for preparing exosome-liposome hybrid structures. Wang et al. used extrusion method to prepare liposome-exosome hybrid structure from bone marrow stromal cells (BMSCs), and successfully encapsulated doxorubicin through ammonium sulfate gradient, and showed better tumor specificity and biocompatibility *in vivo* and *in vitro* (Wang J. et al., 2022). Cheng et al. also reported a membrane fusion technology for cancer immunotherapy by combining engineered exosomes with thermosensitive liposomes and showed substantial accumulation at tumor sites (Cheng et al., 2021).

The exosome-inorganic/organic nanoparticle hybrid system is a relatively novel technique. This hybrid system integrates the advantages of inorganic or organic components to enhance the characteristics of exosomes. Yong et al. loaded DOX into mesoporous silica nanoparticles (DOX-MPS), and found that DOX-MPS can enter tumor cells and cancer stem cell through endocytosis, thereby enhancing the accumulation and infiltration of tumor tissues (Yong et al., 2019). In addition, a bionic nanoparticle platform composed of a metal organic framework was developed and loaded exosomes secreted from MDA-MB-231 cells. This system not only exerts a high loading capacity of foreign proteins (94%) and a high efficiency of exosomes modification (97%), but also shows promising effect of tumor targeting therapy *in vivo* and *in vitro* (Cheng et al., 2018).

Due to the wide range of sources, low cost, stable physicochemical properties, and excellent biocompatibility, Bone marrows (BMs) may become a way for personalized nanodrug delivery in the future. Although some released nanovesicles have been approved, there is still a lot of work to be done (Wang X. et al., 2022), such as the optimal combination of BMs components, large-scale clinical production processes, and the reliability of in human application.

## 9 Future directions

Indeed, MSC-secreted exosomes represent an advanced technology utilized for cancer diagnosis and therapy. The efficacy of these exosomes, concerning sensitivity, specificity, and accuracy, can be influenced by the miRNAs they carry. However, it's worth noting that the current purification methods for exosomes still need continuous refinement and optimization to enhance their performance.

Despite some ongoing clinical trials, various challenges and unanswered questions must be addressed before exosomes can be utilized in humans. In order to produce clinical-grade MSC-derived exosomes without causing any significant toxicity and to achieve consistent and reproducible effects, it is crucial to develop reliable large-scale production methods. Furthermore, ensuring the safety of exosomes is an important issue. This includes preventing contamination with other MSC-derived and maintaining the integrity of exosome content throughout the production process. On the other hand, given the immunomodulatory capacities of MSCs, the extracellular vesicles secreted by MSCs could potentially serve as a viable therapy for graft-versus-host disease (GVHD), which is characterized by acute and chronic severe inflammation in multiple organs. A recent clinical study investigated the effect of human MSCs-derived EVs in acute GVHD mice and showed that MSCs-derived exosomes prolonged the survival of GVHD mice (Fujii et al., 2018). Another study by Zhu et al. explored the safety and effectiveness of aerosol inhalation of EVs derived from human adipose MSCs (hAMSC Exos) in patients with COVID-19. The results showed that lung lesions subsided after continuous inhalation of hAMSC Exos for 5 days, without adverse reactions and well tolerated (Zhu et al., 2022).

Exosomes can encapsulate various miRNAs, some of which may promote the proliferation and aggressiveness of cancer cells (Weng et al., 2021). Currently, producing therapeutic MSCs-derived exosomes involves a substantial workload. To address this, researchers are developing exosome-mimics as an alternative to overcome the production challenges of MSCs-derived exosomes (Lu and Huang, 2020). Another crucial consideration is standardization, which is essential for any therapeutic product.

## 10 Conclusion

This review summarizes the roles of MSCs and MSC-derived exosomes in cancer initiation, progression, diagnosis, and treatment. Absolutely, exosome-based therapeutic strategies show great promise in the fight against cancer. A deeper understanding of the mechanisms of MSCs-derived exosomes and their impact on cancer development is crucial. This knowledge would open up new therapeutic opportunities and innovative strategies to fight cancer.
